# Epidermal closure regulates histolysis during mammalian (*Mus*) digit regeneration

**DOI:** 10.1002/reg2.34

**Published:** 2015-06-09

**Authors:** Jennifer Simkin, Mimi C. Sammarco, Lindsay A. Dawson, Catherine Tucker, Louis J. Taylor, Keith Van Meter, Ken Muneoka

**Affiliations:** ^1^ Division of Developmental Biology, Department of Cell and Molecular BiologyTulane UniversityNew OrleansLouisiana70118USA; ^2^ Department of MedicineLouisiana State University Health Sciences CenterNew OrleansLouisiana70112USA; ^3^ Department of BiologyUniversity of KentuckyLexingtonKentucky40506USA; ^4^ Department of Veterinary Physiology and Pharmacology, College of Veterinary Medicine and Biomedical SciencesTexas A&M UniversityCollege StationTexas77843USA

**Keywords:** Blastema, digit, hypoxia, mouse, regeneration

## Abstract

Mammalian digit regeneration progresses through consistent stages: histolysis, inflammation, epidermal closure, blastema formation, and finally redifferentiation. What we do not yet know is how each stage can affect others. Questions of stage timing, tissue interactions, and microenvironmental states are becoming increasingly important as we look toward solutions for whole limb regeneration. This study focuses on the timing of epidermal closure which, in mammals, is delayed compared to more regenerative animals like the axolotl. We use a standard wound closure device, Dermabond (2‐octyl cyanoacrylate), to induce earlier epidermal closure, and we evaluate the effect of fast epidermal closure on histolysis, blastema formation, and redifferentiation. We find that fast epidermal closure is reliant upon a hypoxic microenvironment. Additionally, early epidermal closure eliminates the histolysis stage and results in a regenerate that more closely replicates the amputated structure. We show that tools like Dermabond and oxygen are able to independently influence the various stages of regeneration enabling us to uncouple histolysis, wound closure, and other regenerative events. With this study, we start to understand how each stage of mammalian digit regeneration is controlled.

## Introduction

The epidermis has long been known to be a major signaling center for limb development and regeneration. During development the apical ectodermal ridge (AER) runs along the distal tip of the developing limb bud and secretes factors that induce proliferation, promote cell migration, and maintain stemness of cells in the underlying mesenchyme (Niswander et al. 1993; Fallon et al. 1994; Cohn et al. 1995; Li & Muneoka 1999). Classic excision experiments show that the AER will not regenerate after removal, and without signaling from the AER distal outgrowth of the limb bud terminates, the underlying mesenchyme differentiates, and ultimately a truncated limb forms (Summerbell 1974; Hu & He 2008). It is clear that interactions between the AER and underlying mesenchyme are necessary for proper limb outgrowth. During the regeneration of limbs in salamanders there is a recapitulation of these epidermal−mesenchymal interactions (Wallace et al. 1981). Following limb amputation, epidermal cells migrate to close the wound within the first 24 h after amputation (Maden 2008). The wound epithelium undergoes a nerve‐dependent transition into a specialized structure called the apical epithelial cap (AEC) (Satoh et al. 2008) which, like the AER, maintains the underlying mesenchyme in an undifferentiated, proliferative state (Christensen et al. 2002; Han et al. 2001). Unlike the AER, the AEC will regenerate when excised; however, if the AEC is damaged by irradiation or if it is continually removed, regeneration is inhibited (Thornton 1957; Lheureux & Carey 1988). The iterated role of the epidermis during limb development and limb regeneration leads one to suspect that the wound epidermis will be an important signaling center during mammalian regeneration as well.

Regeneration in mammals is restricted to the distal tip of the third phalangeal element (P3) (Borgens 1982; Neufeld & Zhao 1995; Han et al. 2008; Fernando et al. 2011). Amputation through the distal region of the P3 element (including nail, epidermis, soft connective tissue, and bone) results in a regeneration response with well‐defined stages. These stages include an initial wound repair response comprising tissue histolysis, inflammation, and epidermal closure that transitions into a rebuilding response to replace the lost tissues (Simkin et al. 2013). Discerning the factors involved in the switch from a degradation state to a regenerative state is integral to understanding mammalian regeneration. In mice, epidermal closure culminates between 8 and 12 days post‐amputation (DPA) (Fernando et al. 2011), a slow and variable response relative to that seen in amphibians (Maden 2008). The histolytic phase, overtly evident by the extensive degradation of stump bone, coincides with the period of epidermal closure (Fernando et al. 2011; Simkin et al. 2013). As the epidermis closes, blastema formation begins and osteolysis ends which points to a potential role of the epidermis in the switch from bone degradation to bone rebuilding. Additionally, studies on epidermal signaling during mouse digit tip regeneration have suggested that the growth and differentiation of mouse P3 epidermal tissues are integral for proper regeneration. Inhibiting nail differentiation by knocking down β‐catenin in keratin 14^+^ cells can change the morphology of the underlying bone and inhibit distal growth of bone following amputation (Takeo et al. 2013). These data suggest that the epidermis is an integral and essential component in P3 regeneration. To better understand the role of the epidermis during mouse digit regeneration, we manipulate the timing of epidermal closure following P3 amputation and measure the effects on both the histolytic and regrowth stages of regeneration.

Methods to promote faster epidermal closure in humans have in the past focused on skin grafting techniques (Fassler 1996). However, in both salamander studies and human observations, the suturing of skin flaps over an amputation wound results in the complete inhibition of any regenerative response (Douglas 1972; Mescher 1976). Instead, to promote faster wound closure we turn to the cyanoacrylates, specifically Dermabond, 2‐octyl cyanoacrylate. Cyanoacrylates are liquid monomers that polymerize upon contact with air and form a flexible skin adhesive. In general, use of cyanoacrylates has led to improved wound healing as measured by faster wound closure, lower rates of infection, and improved physical appearance of scars (Singer & Thode 2004; Nipshagen et al. 2008; Singer et al. 2008; Wachter et al. 2010). Dermabond specifically has been shown to have several beneficial wound healing characteristics including a low water vapor transmission rate, a high tensile strength, and anti‐microbial properties (Nitsch et al. 2005; Singer et al. 2008; Gooch 2010). The application of Dermabond to skin wounds has been shown to reduce tissue death (Yamamoto & Kiyosawa 2012), limit the inflammatory response (Singer et al. 2008; Wachter et al. 2010), change the oxygen environment (Winter 1977), and alter the hydration state of the tissue (Winter 1963; Singer et al. 2008). However, the molecular effects of Dermabond on the wound environment have not been extensively studied and the mechanism of action underlying Dermabond's fast wound closure ability is not well documented. Here we utilize Dermabond to manipulate epidermal closure following P3 amputation and show that Dermabond induces early wound closure, reduces bone degradation, and modifies endogenous regenerative abilities. Furthermore, we demonstrate that local hypoxia promoted by Dermabond treatment is an integral part of accelerated wound closure and diminished histolysis. With these studies, we propose a mechanism by which epidermal closure controls the histolytic stage of regeneration and subsequently changes the process of bone regrowth during digit regeneration.

## Results

### Dermabond accelerates wound closure during digit regeneration

**Figure 1:**
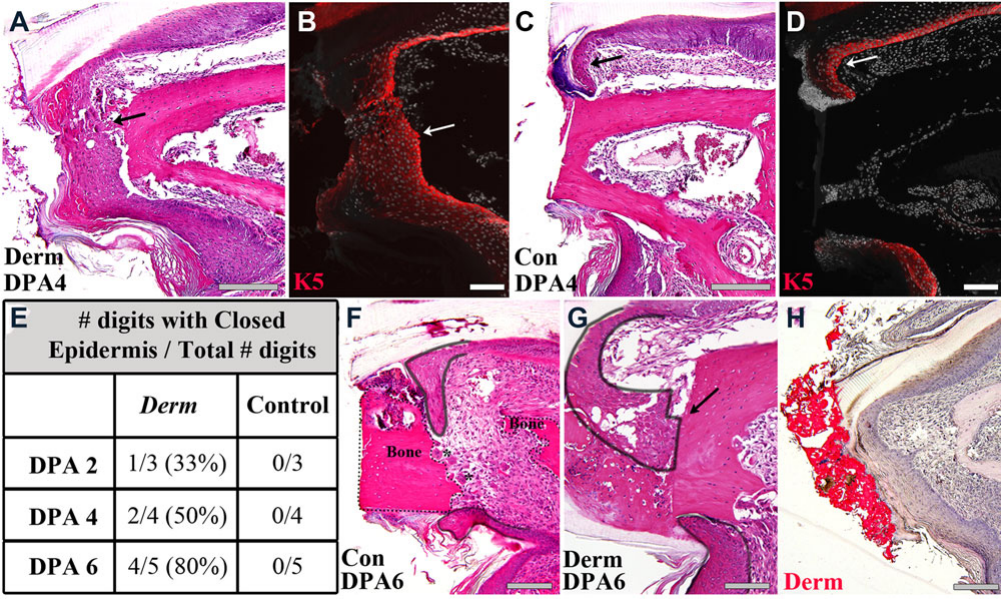
Dermabond treatment promotes epidermal closure following P3 amputation. (A) H&E staining of a Dermabond‐treated digit and (B) keratin 5 expression of a serial section shows the epidermis is closed at DPA 4 and directly touches the distal edge of the bone stump (arrow). (C) H&E staining of a control digit and (D) keratin 5 expression of a serial section shows the epidermis is still open at DPA 4 and a fold forms at the distal edge of the epidermis (arrow). Representative samples are shown. Scale bars: 200 μm in (A), (C), and 100 μm in (B), (D). (E) Timing of complete epidermal wound closure occurs between DPA 2 and DPA 6 in Dermabond‐treated digits and after DPA 6 in all control digits tested. *N* = 12, control and Dermabond‐treated. (F) Epidermal closure in control digits at DPA 6 transects the bone stump (bone outlined with dotted black line). (G) Epidermal closure in Dermabond‐treated digits at DPA 6 occurs across the distal edge of the bone stump (epidermis outlined in solid black). (H) Oil Red O staining of a Dermabond‐treated digit at DPA 6 identifies the cyanoacrylate (red) and shows that epidermal closure occurs directly under the Dermabond covering. Scale bars 100 μm.

Dermabond was used as a wound dressing immediately following digit tip amputation. Dermabond was applied to amputated digits of the left paw, and amputated digits of the right paw were left untreated as a control. To measure wound closure timing, Dermabond‐treated and control digits were collected at DPA 2, 4, or 6, and fixed, processed, and serially sectioned. Two transverse serial sections were collected every 40 μm through the entire digit. Hematoxylin and eosin (H&E) staining (Fig. 1A, C) and the keratinocyte specific marker keratin 5 (Fig. 1B, D) were used to identify the epidermis. Digits were calculated as having a completely closed epidermis if dorsal and ventral epidermal edges met in all sections of the digit. The timing to epidermal closure following Dermabond treatment was significantly faster; however, like control digits it was highly variable (Fernando et al. 2011). As early as 2 DPA, 33% (1/3) of the digits analyzed were completely closed. By 4 DPA 50% of digits were closed (Fig. 1A, B) and at 6 DPA the majority of digits (4/5) displayed complete wound closure (Fig. 1E). Consistent with a previous study in which wound closure was completed between 8 and 12 DPA (Fernando et al. 2011), all 12 time‐matched control wounds analyzed remained open at all times analyzed (Fig. 1C−E).

Dermabond‐treated amputations also demonstrate a difference in the dynamics of the epidermal closure response. In Dermabond samples, epidermal cells are observed making direct contact with the distal edge of the bone stump as wound closure commences (Fig. 1A, B, arrow). In control samples, epidermal cells do not migrate across the amputated bone stump but contact the stump at a more proximal level. In controls, the epidermis forms a characteristic distal fold that gives the appearance of distal‐ward expansion of the epidermal sheet with the free edge anchored onto the bone stump (Fig. 1C, D, arrow). A closer histological analysis of control digits at DPA 6 shows epidermis invading regions where bone stump erosion is associated with the presence of osteoclasts, thus creating the appearance of a secondary stump amputation (Fig. 1F). In contrast, the single Dermabond‐treated digit sample at DPA 6 that failed to complete epidermal closure shows that the dorsal epidermis is in direct contact with the amputated stump bone, whereas the ventral epidermis appears to be anchored to the lateral stump (Fig. 1G, black outline, arrow). The histological evidence suggests that Dermabond is influencing the adhesive properties of the healing epidermis allowing it to migrate across the amputated bone surface. Digit regenerates processed for histology to maintain the Dermabond wound dressing and stained with Oil Red O show that the epidermis closes beneath the dressing and indicate that Dermabond is not incorporated into the amputation wound itself (Fig. 1H). Because the Dermabond is subsequently sloughed off, its physical effect on the regeneration process is transient and restricted to early stages associated with wound closure.

**Figure 2:**
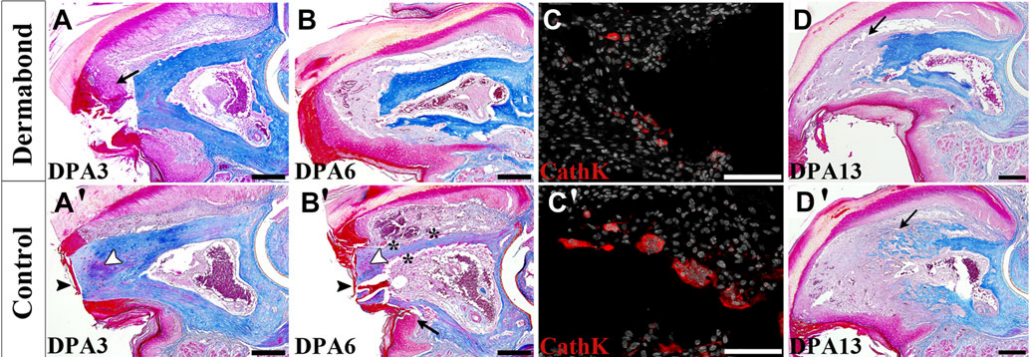
Mallory trichrome stained samples of Dermabond‐treated digits (A, B, D) and control digits (A′, B′, D′) at DPA 3, 6, and 13 show distinct histological differences during the first 13 days of regeneration. (A) Dermabond‐treated digits show the epidermis has started to close prematurely at DPA 3 (black arrow). (A′) Control digits at the same time point have open epidermis and blood filled osteocyte lacunae (white arrowhead). (B) By DPA 6 Dermabond‐treated digits show a collection of cells at the distal tip of the bone stump, and osteoclast resorption pits are noticeably absent. (B′) DPA 6 control digit shows osteoclast resorption pits (asterisks) and the beginning of epidermal wound closure on the ventral side of the digit (black arrow). (C) Immunofluorescent staining for the osteoclast‐specific enzyme cathepsin K (CathK, red) shows the presence of osteoclasts as small cells with single nuclei in Dermabond‐treated digits. (C′) Staining for cathepsin K in control digits shows large multi‐nucleated osteoclasts. Cathepsin K, red; DAPI, grey. (D) Dermabond‐treated digits also show a distal blastema, and new bone growth is observable as ribbons off the original bone stump (black arrow) at DPA 13. (D′) Control digits form a blastema, and new trabecular bone growth is observable by DPA 13 (black arrow). Scale bar: 200 μm in (A), (B), (D), and 100 μm in (C).

Mallory trichrome staining was used to investigate histological changes induced by Dermabond during wound closure and blastema formation. At DPA 3 there is clear evidence that the epidermis of Dermabond‐treated regenerates is actively closing the amputation wound over the bone stump, whereas the amputation stump in control regenerates is devoid of epidermal cells (Fig. 2A, A′). At this time point the control stump is covered by a blood clot and blood islands are observed in the osteocyte lacunae of the distal bone stump suggestive of an injury response deep within the stump bone (Fig. 2A′, white arrowheads). Similar blood islands were absent in the bone stump of Dermabond‐treated digits (Fig. 2A). At 6 DPA wound closure is complete in Dermabond‐treated regenerates and there is an accumulation of a blastema between the wound epidermis and the bone stump (Fig. 2B). The cortical bone of the digit stump appears largely intact in Dermabond‐treated samples compared to control regenerates at 6 DPA which are characterized by many erosion pits lining the endosteal and periosteal layers (Fig. 2B, B′). Immunostaining for the osteoclast‐specific enzyme cathepsin K (Li et al. 1995) reveals the presence of osteoclasts in both Dermabond‐treated and control digits; however, Dermabond treatment was associated with small single‐nucleated osteoclasts (Fig. 2C) whereas the erosion pits in control digits contained large, multi‐nucleated osteoclasts (Fig. 2C′). By DPA 13 bone regrowth has commenced in both treated and untreated digit samples (Fig. 2D, D′); however, the organization of newly deposited bone appears to be quite different (see below). Together these data provide evidence that Dermabond promotes precocious epidermal closure and blastema formation after digit tip amputation. The data also suggest that the bone degradation phase of the regenerative response is inhibited by Dermabond treatment.

### Dermabond treatment an inhibits bone degradative

**Figure 3:**
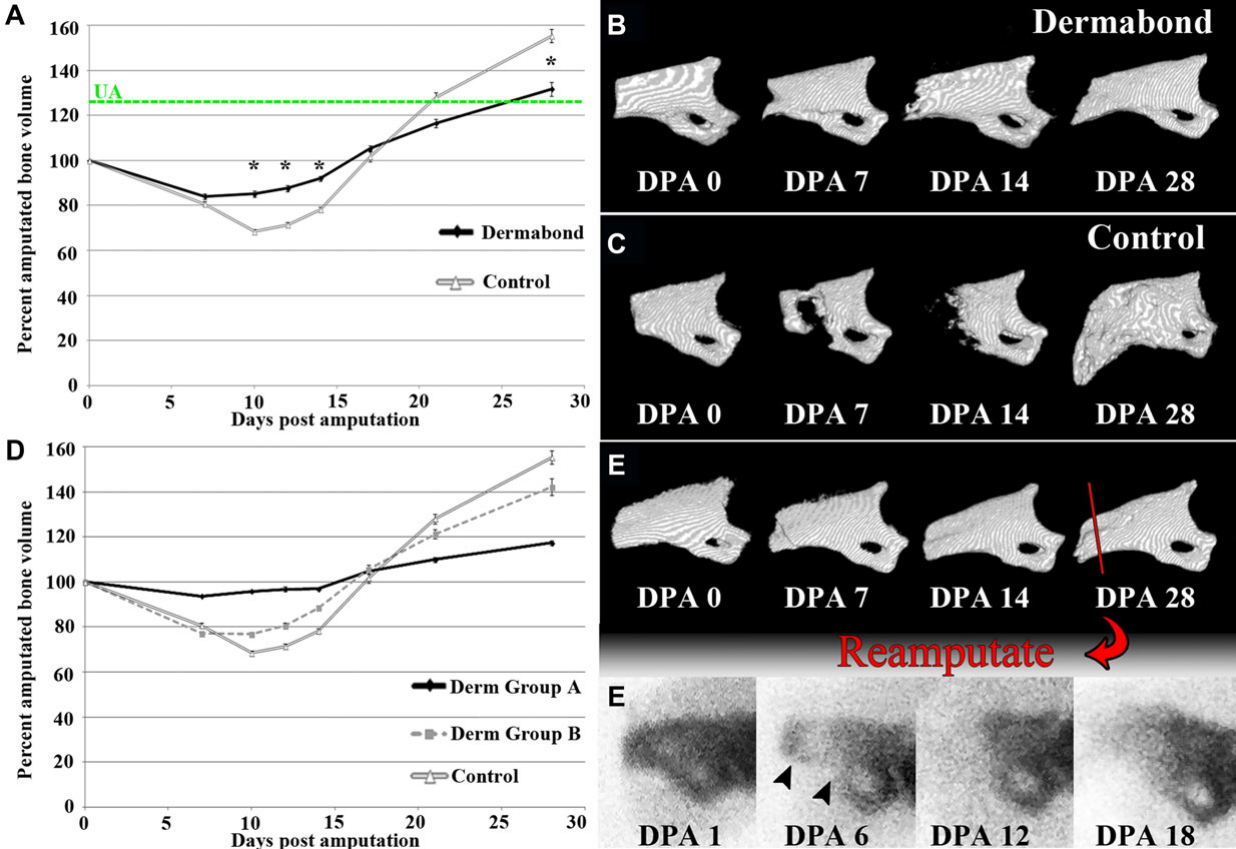
Dermabond treatment attenuates bone degradation during regeneration. (A) Dermabond‐treated digits show reduced bone degradation at DPA 7−12 (*N* = 12 mice, *N* = 24 digits). Samples were analyzed for bone growth using μCT. Data are normalized to initial DPA 0 bone volume and shown as a percentage. Error bars represent SEM. The initial unamputated (UA) bone volume (green dotted line) averages 125% of the amputated bone volume. 3D renderings of representative samples of Dermabond‐treated (B) and control (C) digits during regeneration illustrate the loss of a degradation stage in Dermabond‐treated digits. (D) The loss of degradation following Dermabond treatment is variable. 45% of digits treated display no loss of bone volume (Derm Group A, *n* = 11). The other 55% of digits treated are comparable to control digits (Derm Group B, *n* = 13). (E), (F) Re‐amputation of DPA 28 Dermabond‐treated digits after regeneration show return of the degradation phase during subsequent regeneration events. (E) 3D renderings show a regenerated Dermabond‐treated digit prior to re‐amputation from DPA 1 to DPA 28. (F) Representative X‐ray images of the same digit shows return of bone degradation after re‐amputation and the subsequent regeneration event—DPA 1, 6, 12, and 18. *N* = 4, Representative sample shown.

To characterize the regenerative response in Dermabond‐treated digits, we tracked bone volume changes over time with micro‐computed tomography (μCT) scans. Bone morphology and volume changes during digit tip regeneration identify two distinct phases of the regenerative response: (1) an initial bone degradation phase characterized by bone erosion and loss in bone volume, and (2) a bone rebuilding phase in which new bone is laid down in a proximal to distal pattern and volume measurements reveal a characteristic overshoot in bone formation (Fernando et al. 2011; Sammarco et al. 2014). Thus, the regenerative process can be characterized by an early loss in bone volume that transitions into a bone building phase. Volume measurements show that by DPA 10 control digits lose an average of 32% of their amputated volume whereas Dermabond‐treated digits lose significantly less volume (15%, *P* = 0.0001). Three‐dimensional (3D) renderings of μCT data provide examples for changes in bone morphology during the regenerative response in control (Fig. 3C) and Dermabond‐treated (Fig. 3B) digits. These 3D renderings point to clear differences in the amount and proximal−distal level of bone resorption associated with Dermabond treatment. Closer examination of this data set shows that approximately half (11/24; 45%) of the Dermabond‐treated digits have a phenotype shown in Figure 3B; regenerating digits display little notable loss of bone volume (Fig. 3D, Derm Group A). The amount and pattern of bone loss of the remaining digits (13/24; 55%) was similar to untreated control digits (Fig. 3D, Derm Group B). While this clear bifurcation of the Dermabond‐induced response is remarkable, it is unclear at this time how it might be linked to the established effect of Dermabond on wound closure rate. We note that the Dermabond dressing appears intact on all digits based on gross inspection, although it is possible that partial damage to the dressing could account for digits that appear similar to control digits. Nevertheless, the overall effect of the Dermabond dressing on bone volume loss after amputation is both dramatic and statistically significant.

Bone volume measurements also demonstrate that Dermabond‐treated digits are able to regenerate bone back to unamputated levels by DPA 28 indicating that Dermabond treatment is not inhibitory for a regenerative response. However, compared to control regenerates, there is a clear and significant difference in the response. Dermabond‐treated regenerates lack the characteristic volume overshoot typically displayed by untreated control digits. We note that both control and Dermabond‐treated regenerates transition to the bone rebuilding phase between DPA 14 and DPA 17, suggesting that despite an early onset of blastema formation (see Figs 1H and 2B) the timing of overt bone growth during regeneration does not appear to be influenced by Dermabond treatment (Fig. 3A).

To determine if the effect of Dermabond on bone loss during the regenerative response is transient or permanent, we performed re‐amputation experiments on Dermabond‐treated digits. In these experiments, digits were amputated, treated with Dermabond, and imaged with μCT during the regeneration process. Digits that showed a loss of less than 10% amputated bone volume (Derm Group A) and had regained bone volume to an unamputated level by DPA 28 (Fig. 3E) were re‐amputated. No Dermabond was applied after re‐amputation and bone changes were monitored with standard X‐ray analysis (Fig. 3F). All digits tested (*n* = 4) exhibited a degradation and regrowth response following re‐amputation that paralleled untreated control regeneration. The bone degradation response cleaves the stump bone into two pieces (Fig. 3F, arrows), the distal bone fragment is lost, and newly regenerated bone is laid down in a proximal to distal pattern. Dermabond‐treated digits therefore maintain the ability to progress through a normal regenerative response when no dressing is applied, indicating a transient effect of Dermabond treatment.

### Dermabond permits a modified bone regeneration response via a blastema intermediate

**Figure 4:**
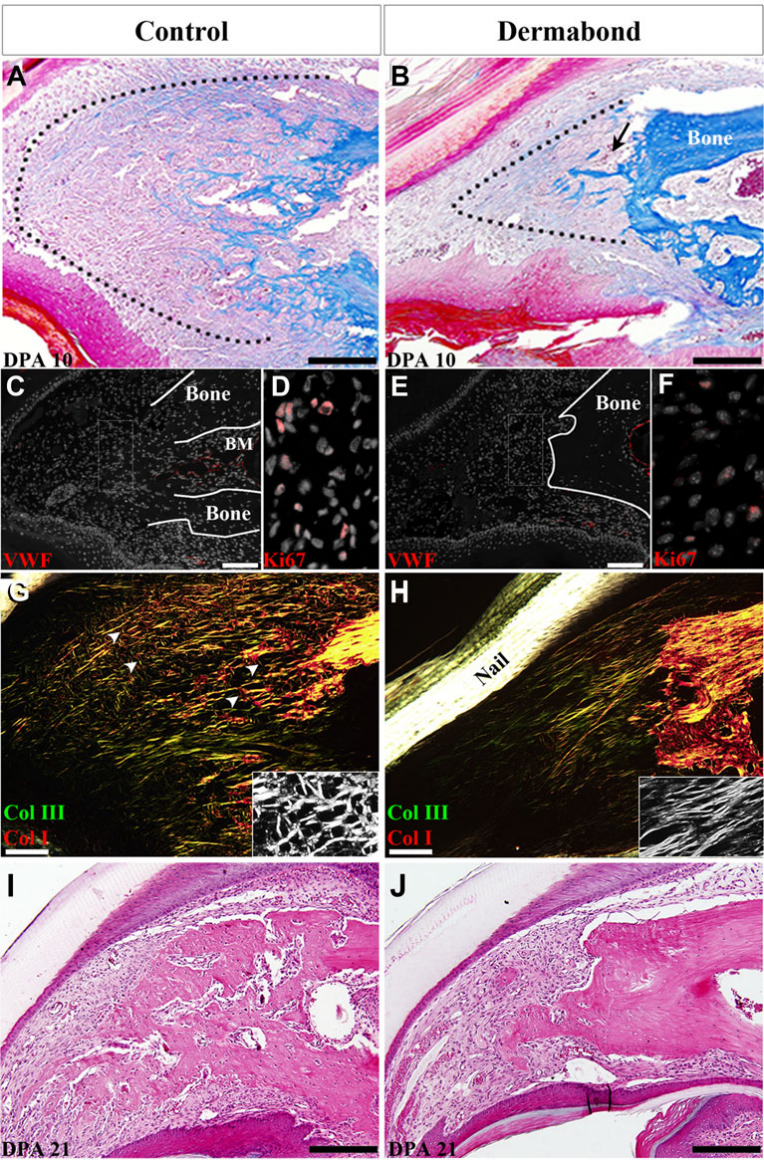
Mallory trichrome staining at DPA 10 shows both (A) a control digit and (B) a Dermabond‐treated digit with the blastema area outlined. Dermabond‐treated digits show ribboned bone growth (black arrow). Scale bar 200 μm. (C) Control digits are negative for von Willebrand factor 8 (VWF) staining in the blastema. Positive staining can be seen in the marrow region. Bone stump outlined in white. Scale bar 100 μm. (D) Control blastema shows Ki67 positive cells. (E) Dermabond‐treated digits show the blastema is negative for VWF staining. Red, VWF; grey, nuclei. Bone stump outlined in white. Scale bar 100 μm. (F) High magnification of boxed rectangle in (D) in a serial section of the same Dermabond‐treated digit shows Ki67 staining for proliferation. Red, Ki67; grey, nuclei. (G) Polarized light micrograph of the control sample at DPA 10 shows woven collagen fibers and collagen I and collagen III localization. Col III, green; Col I, red. (H) Polarized light micrograph of DPA 10 Dermabond‐treated sample shows collagen fibers aligned parallel to each other and the presence of collagen III (green) and collagen I (red/yellow). Scale bar 200 μm. Insets show a monochrome version of each picture to better visualize the collagen fiber orientation. (I), (J). H&E staining of DPA 21 samples of (I) a control digit and (J) a Dermabond‐treated digit which shows fewer trabecular spaces and less bone overgrowth than a control digit at the same time point. Scale bars 200 μm.

Despite the fact that Dermabond limits the degradation stage of regeneration, bone growth is still observed and treated digits regenerate to a volume comparable to unamputated levels (Fig. 3A). We use histological and immunohistochemical analysis to determine if regeneration following Dermabond treatment progressed through a blastema intermediate similar to control digits. In control digits, at the time of the blastema stage (DPA 10−12), an accumulation of condensed, morphologically indistinct, proliferative cells is observed at the distal tip of the amputated bone (Fig. 4A, dotted line). This population of cells is avascular, positive for proliferation markers, and will go on to form the structures of the missing digit tip (Fernando et al. 2011) (Fig. 4C, D). Much like the blastema of a regenerating axolotl limb (Makanae & Satoh 2012) or spiny mouse ear (Seifert et al. 2012), the blastema of the mouse digit also shows lower levels of mature matrix proteins like collagen I and higher levels of transient matrix proteins like collagen III (Fig. S1). Similar to control digits, Dermabond‐treated digits show an accumulation of a condensed, morphologically indistinct population of cells (blastema) at the cut edge of P3 beneath the wound epidermis (Fig. 4B, dotted line). This area is visibly smaller than the blastema of control digits (Fig. 4A) at this same time point. The Dermabond‐treated digit blastema is largely devoid of cells staining positive for the endothelial cell marker von Willebrand factor VIII (Fig. 4E) consistent with previous studies of regenerating digit blastemas and indicative of the avascular character of blastemas in general (Peadon & Singer 1966; Yu et al. 2014). Cells within both control and Dermabond‐treated digit blastemas are also proliferating based on immunostaining for the proliferation‐specific protein Ki67 (Fig. 4F). Additionally, picrosirius red staining viewed under polarized light microscopy (Montes & Junqueira 1991; Rich & Whittaker 2005) reveals collagen III (green) and collagen I (red) deposition at DPA 10. Both in control (Fig. 4G) and Dermabond digits (Fig. 4H), thin collagen III fibers are observable in the distal blastema area while collagen I fibers are more prevalent in the proximal blastema area as new bone is deposited. We therefore conclude that Dermabond‐treated digits are able to regenerate through a blastema intermediate much like control digits.

Closer examination of collagen fiber orientation in picrosirius red stained samples viewed with polarized light microscopy (Shapiro 2008) reveals a difference in the regeneration process of Dermabond‐treated digits versus controls. By DPA 10, new bone forms off the distal edge of the amputated bone stump in Dermabond‐treated samples (Fig. 4B, arrow). Qualitative examination of polarized light micrographs reveals collagen fibers oriented parallel to and contiguous with the original cortical bone stump of these digits (Fig. 4H, inset). The parallel orientation of collagen fibers is characteristic of lamellar bone formation and a higher resistance to tensile forces (Bromage et al. 2003; Shapiro 2008). Collagen fibers in control digits, on the other hand, are deposited in a woven pattern at the same time point (Fig. 4G). The crosshatched pattern can be observed at DPA 10 before new bone begins to ossify (Fig. 4G, arrows and inset). The change in bone growth becomes more obvious at later time points. Dermabond‐treated digits show bone growth without many trabecular spaces (Fig. 4J) whereas control digits at this same time point show considerable trabecular spacing throughout the regenerated digit tip (Fig. 4I). This change from woven bone formation in control digits to lamellar bone formation in Dermabond‐treated digits is suggestive of an alteration in the mechanics of bone patterning during the regeneration response.

### Dermabond promotes an hypoxic event during regenerative

**Figure 5:**
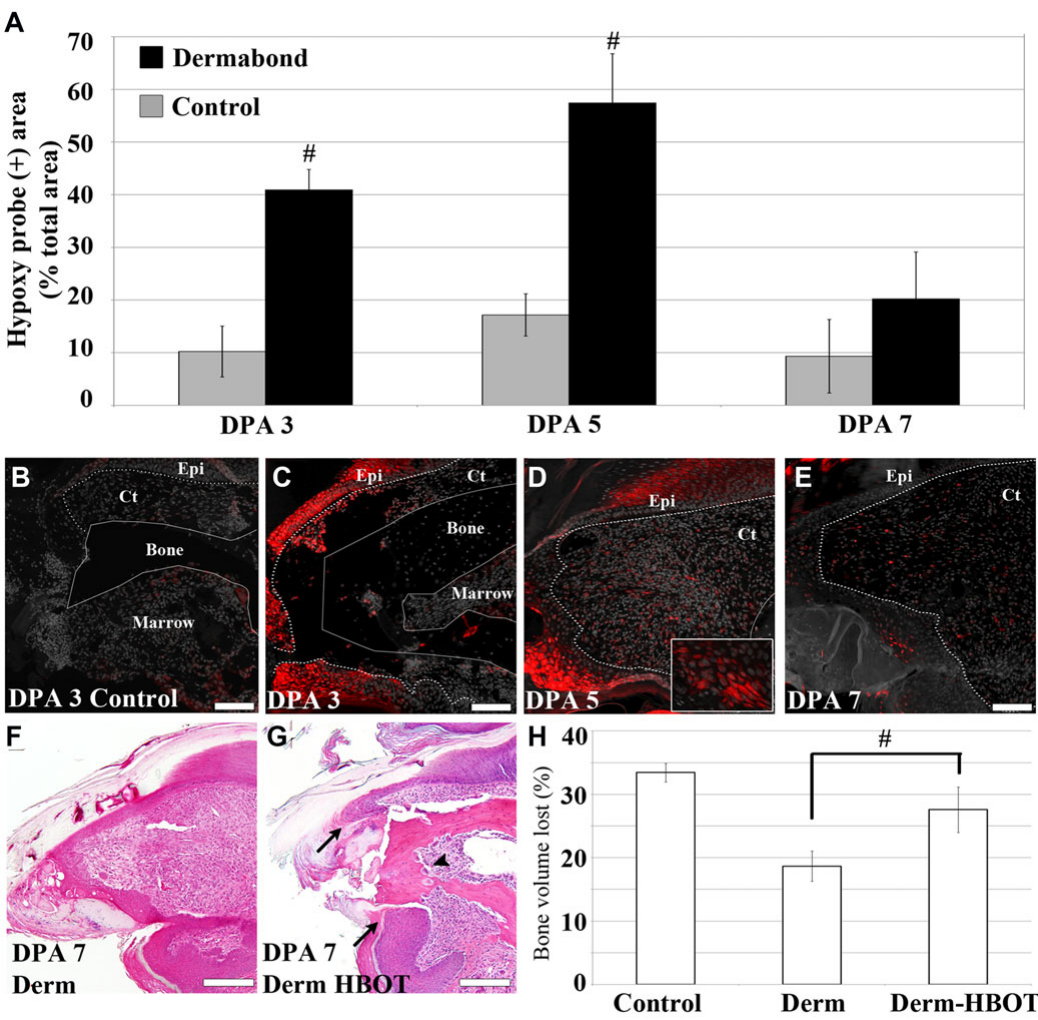
(A) Oxygen profiling of the epidermis in control and Dermabond‐treated digits using Hypoxyprobe (<1.3% oxygen) indicates relatively hypoxic microenvironments in the epidermis at DPA 3 and 5 in Dermabond‐treated digits. The anti‐Hypoxyprobe stained cells were selected and plotted as cell counts versus DAPI staining. Percentages of cells per total cellular area are shown in the bar graph. ^#^ *P* < 0.05. (B) Analysis of the hypoxic signal in control digits at DPA 3 shows few areas of hypoxia. (C) In comparison Dermabond‐treated digits at DPA 3 show hypoxic epidermis. The dotted outline delineates epidermis and underlying bone or connective tissue. (D) DPA 5 shows a hypoxic epidermis and a migratory phenotype of hypoxic cells (inset). (E) Dermabond‐treated digits show a decrease in hypoxia in the epidermis by DPA 7. *N* = 5 with representative samples shown. Red, Hypoxyprobe; grey, nuclei; Epi, epidermis; Ct, soft connective tissue. Scale bar 100 μm. (F) H&E staining of a Dermabond‐treated digit at DPA 7 showing complete wound closure. (G) H&E staining of a Dermabond‐treated digit at DPA 7 after daily HBOT treatment showing open epidermis (arrows). *N* = 3 mice, seven digits with representative sample shown. Scale bar 200 μm. (H) Control, Dermabond‐treated and Derm‐HBOT‐treated samples analyzed for loss of bone volume show that Derm‐HBOT‐treated samples have bone degradation levels comparable to control digits. Digits were tracked with μCT for 21 days and the maximum amount of bone volume lost was quantified. Data are normalized to the initial DPA 0 amputated bone volume. *N* = 4 mice, 14 digits. Error bars represent SEM.

Given that oxygen levels are known to influence the rate of wound closure (Winter 1963; Li et al. 2007; Mace et al. 2007; Botusan et al. 2008; Zimmermann et al. 2014) we utilized Hypoxyprobe (2‐pimanidiazole‐HCl) to evaluate hypoxic microenvironments during the wound closure time period following Dermabond treatment. Hypoxyprobe is stabilized in areas of less than 1.3% oxygen tension. Hypoxyprobe was utilized at 0, 3, 5 or 7 DPA (Fig. 5) and quantified by calculating the percent fluorescent positive signal in the epidermis, connective tissue, or bone marrow normalized to total DAPI^+^ (4',6‐diamidino‐2‐phenylindole) area. Control digits show minimal Hypoxyprobe signal in the epidermis at the stages analyzed (Fig. 5A, B). In contrast, Dermabond‐treated digits show significantly greater regions of hypoxic epidermis compared to controls at DPA 3 and 5 (Fig. 5A−C). Comparison of Hypoxyprobe signal in the bone marrow and connective tissue showed no significant difference between Dermabond and control samples at these early time points (Fig. S2). By DPA 7, the hypoxic area of the Dermabond‐treated epidermis is comparable to that of control digits, and a significant difference is evident in the connective tissue compared to controls (*P* = 0.002) (Fig. S2). Both groups show an increase in bone marrow signal from DPA 3 to DPA 7 consistent with our previous findings (Sammarco et al. 2014) (Fig. S2). Representative images display the change in the hypoxic microenvironment of Dermabond‐treated digits over the time course of epidermal closure (Fig. 5C−E**)**. Hypoxyprobe staining is prominent in the epidermis at DPA 3 (Fig. 5C, epidermis and bone delineated by a white dashed line for orientation) and DPA 5 (Fig. 5D). Additionally hypoxic cells of the wound epidermis appear to have a migratory phenotype with flattened and elongated cell bodies (Fig. 5D, inset). Hypoxyprobe staining is less evident in the epidermis of Dermabond‐treated digits at DPA 7 and more apparent in the underlying connective tissue area (Fig. 5E). Thus, Dermabond‐treated digits display a dynamic hypoxic microenvironment at early time points during regeneration with the majority of the hypoxic area localized to the epidermis.

To investigate whether the low oxygen events observed in Dermabond‐treated digits are causative in accelerated epidermal closure and/or the subsequent loss of bone degradation, we applied pulses of hyperbaric oxygen treatment (HBOT) during regeneration and monitored wound closure and bone degradation. Mice received HBOT at 2.4 atmospheres absolute (ATA), twice a day for 90 min over the course of regeneration. Digits were analyzed histologically for epidermal closure and bone volume was tracked with μCT to record maximal bone loss. Epidermal closure in all Dermabond‐treated digits is complete by DPA 7 (Figs 1E and 5F). When treated with hyperbaric oxygen, 100% of digits (*n* = 7) failed to close the epidermis at DPA 7 (Fig. 5G, arrows), thus showing that hyperbaric oxygen counteracts the Dermabond effect on epidermal closure rate. Similarly, Dermabond‐treated HBOT digits (Derm‐HBOT) exhibit an average bone volume loss that is significantly greater than that with Dermabond treatment alone (*P* = 0.039) and is not significantly different from untreated control digits (*P* = 0.078) (Fig. 5H). These data show that HBOT can reverse the effects of Dermabond treatment, slowing wound closure and increasing the amount of bone degradation, thus providing evidence that the Dermabond effect on digit regeneration is mediated at least in part by oxygen.

## Discussion

The stages of mouse digit regeneration have a distinct order. Following amputation, we observe (1) an initial clotting phase, (2) an inflammatory response, (3) a histolytic response (primarily associated with the activation of osteoclasts and resulting in bone degradation), (4) epidermal closure with the formation of a wound epidermis, (5) blastema formation, and (6) redifferentiation of bone and surrounding tissue (Simkin et al. 2013). While the events associated with a mammalian regenerative response are generally similar to those of more conventional regeneration models (e.g., the salamander limb), the sequence of events differs dramatically. Following limb amputation in the axolotl, an initial clotting event is followed by rapid epidermal closure within the first 24 h (Maden 2008). After wound closure, a peak in the inflammatory response is observed (Godwin et al. 2013) and the stump tissues undergo a histolytic response that culminates in the formation of the limb blastema. In both amphibian and mammalian regeneration models, a key issue is to understand the interdependence of these regenerative phases and how any one phase is linked to a successful transition to the next. In the current study we are able to enhance the rate of wound closure in the regenerating mouse digit tip by using Dermabond as an initial wound dressing. Dermabond treatment enhances hypoxia specifically in epidermal cells and also causes a significant reduction in the histolytic phase of the regenerative response. While Dermabond treatment influences early regeneration events, it does not inhibit blastema formation or the timing of overt redifferentiation of bone and surrounding tissue. These data suggest that epidermal closure of the amputation wound interfaces with the histolytic phase but does not significantly alter the timing of subsequent regenerative phases.

### Epidermal wound closure and hypoxia

Previous studies with Dermabond treatment have clearly documented the ability of this product to increase epidermal closure time and improve the physical appearance of the scar (Singer et al. 2008; Yamamoto & Kiyosawa 2012). Occlusive dressings have been used to increase the rate of wound closure in multiple clinical and laboratory settings. The ability of occlusive dressings to improve wound healing is attributed to several factors including retention of moisture and reduction of inflammation. We cannot rule out that these factors may also play a role following Dermabond treatment during wound closure in digit regeneration (Winter 1963; Singer et al. 2008; Wachter et al. 2010). However, several studies point to the fact that the more impermeable a dressing is to oxygen diffusion the better the rate of wound closure (Winter 1963; Tandara & Mustoe 2008), and that synthetic dressings tend to create a hypoxic wound environment (Varghese et al. 1986). Our study supports this hypothesis: Dermabond treatment is followed by an early and enhanced hypoxic event in the epidermis that is associated with accelerated wound closure. Elimination of this hypoxic event with hyperbaric oxygen slows the rate of wound closure suggesting that oxygen availability is linked to the rate of epidermal migration in vivo. Our findings add to a growing body of evidence showing that epidermal migration during wound closure is regulated by oxygen levels (Mace et al. 2007; Botusan et al. 2008; Zimmermann et al. 2014). Hypoxia is thought to promote keratinocyte migration indirectly by favoring extracellular matrix remodeling through the reduced expression of elastin, activation of matrix degrading enzymes like metalloproteinases, and stimulation of new collagen synthesis (Boraldi et al. 2007), and directly through the upregulation of specific integrins (Kalucka et al. 2013). In sum, the data suggest that epidermal cell migration can be controlled by regulating oxygen availability, and this conclusion is likely to be important for the design of regenerative medicine therapies.

### Bone degradation

Digit tip regeneration in adult mice is characterized by a histolytic phase that is associated with enhanced osteoclast activity that results in an injury induced secondary amputation (Fernando et al. 2011). During this process the epidermis does not migrate over the amputated bone stump and eventually closes through the eroded bone stump. An association between epidermal closure and bone histolysis has also been noticed in axolotl limb regeneration but this interaction has not been studied in detail. In the regenerating limb, if the stump bone is not trimmed the protruding bone creates a physical impediment for the epidermis and wound closure is delayed until the stump bone is degraded (Maden 2008). It is therefore likely that a link between epidermal closure and histolysis is not unique to mammalian regeneration but is observed in other regeneration models. Bone degradation is typically associated with osteoclast activity, and we have previously shown that enhancing oxygen availability with HBOT promotes the activity of osteoclasts during regeneration (Sammarco et al. 2014). Here we demonstrate that the use of Dermabond as a wound dressing results in an abnormally hypoxic microenvironment that is associated with a reduction in osteoclast activity. These data show that oxygen availability regulates not only epidermal migration and wound closure, but also osteoclast activity and bone histolysis. The strict correlation between oxygen effects on wound closure and bone histolysis and the timing of wound closure with the end of the histolytic phase in the endogenous regenerative response is suggestive that they are intricately linked. Since hyperbaric oxygen does not enhance bone degradation in unamputated digits (Sammarco et al. 2014), the effect of oxygen on osteoclast activity appears to be linked to other known modifiers of the wound microenvironment, for example the wound epidermis. This observation may help to explain discrepancies observed in *in vitro* studies in which enhanced osteoclast activity is reported in both high and low oxygen conditions (Arnett et al. 2003; Fukuoka et al. 2005; Yamasaki et al. 2009; Arnett 2010). We propose that one function of the wound epidermis is to inhibit osteoclast activity thereby signaling a transition from the histolytic phase into the blastema phase of regeneration.

Despite a significant reduction of histolysis in Dermabond‐treated digit amputations, a regenerative response is still observed. Bone growth in the absence of bone degradation has been described in several mammalian models of bisphosphonate‐treated bones (Mashiba et al. 2005; Marx 2014). Treatment with bisphosphonates reduces the amount of bone degradation in patients with osteoporosis by promoting osteoclast apoptosis. Bisphosphonate‐treated patients maintain the ability to form new bone and increase bone mineral densities (Marx 2014); however, the loss of osteoclast remodeling can lead to excessively brittle bone more prone to micro‐fractures. Arguably better treatments for osteoporosis focus on shifting the balance of bone turnover from degradation to regrowth without the complete loss of osteoclast activity, making the turnover system more permissive for the repair of damaged bone (Fonseca 2008). Since Dermabond treatment limits osteoclast activity without eliminating the osteoclasts, the digit regeneration model represents a useful model for dissecting the in vivo relationship between osteoclasts, osteoclast activity and osteoblast activation as it pertains to bone regrowth.

### Retention of regenerative abilities

One of the remarkable findings of the Dermabond effect is that the regenerated digit tip has a skeletal structure that closely mimics the amputated digit tip. Adult digit tip regeneration in mice is characterized by the replacement of amputated cortical bone with woven bone, and bone volume measurements show that the regenerated bone consistently overshoots the pre‐amputation volume (Fernando et al. 2011; Sammarco et al. 2014). Despite the fact that the regenerative response results in a functional digit tip, this overshoot suggests a defect in skeletal patterning associated with the mammalian regenerative response. Similarly, regeneration following hole punch of ears in select strains and species of mice is reported to be defective compared to uninjured tissue (Metcalfe et al. 2006; Seifert et al. 2012) and these observations suggest that imperfections linked to the evolution of regenerative responses in mammals are the norm rather than the exception. The overall effects of Dermabond treatment on the regeneration response are (1) to enhance the rate of wound closure, (2) to reduce the histolytic response, (3) to reduce the size of the digit blastema, and (4) to regenerate new bone tissue that does not have the histological appearance of woven bone and does not overshoot the initial bone volume. Thus, Dermabond treatment appears to minimize the skeletal patterning defect typically associated with the endogenous regeneration response, and suggests that this simple wound dressing improves the overall quality of the regenerative response.

One of the outcomes of the bone histolytic response is the exposure of the bone marrow region, and the cells within, to the amputation wound site. The reduction in bone histolysis in Dermabond‐treated digits has two obvious effects. First, because the terminal phalangeal bone tapers to a pointed tip, the amputation wound is smaller than the wound that forms following histolysis of the distal bone stump. As a consequence the wound epidermis that forms is smaller as is the region that forms the digit regeneration blastema. Second, because the distal bone stump remains intact and the marrow region is not compromised, the involvement of bone marrow derived cells in blastema formation and the regenerative response is potentially reduced. We propose that these two anatomical effects caused by Dermabond treatment modify the microenvironment within which the digit blastema forms and are causally linked to morphogenetic interactions associated with the regeneration of new bone tissue. Collagen fiber alignment has been shown to be a product of osteoblast orientation, and mechanical forces in the environment play a key role in orienting osteoblasts (Shapiro 2008). Woven bone forms from mesenchymal osteoblasts that display a random interlacing orientation, whereas osteoblast orientation that is highly organized on the surface of structurally stable bone results in lamellar bone (Shapiro 2008). The qualitative difference in collagen fiber alignment that we observe, interwoven versus parallel, in untreated versus Dermabond‐treated blastemas, respectively, is consistent with a change in the cellular composition of the blastema and a modification of the mechanical forces that influence morphogenesis within the blastema microenvironment. Both of these changes are predicted by a reduction in bone histolysis resulting from the use of Dermabond for wound coverage.

## Materials and Methods

### Amputations and animal handling

Adult 8‐week‐old female CD1 mice were obtained from Charles River Laboratories (Wilmington, MA). Mice were anesthetized with 1−5% isoflurane gas with continuous inhalation. The second and fourth digits of both hind limbs were amputated at the P3 distal level as described previously (Fernando et al. 2011; Simkin et al. 2013). Digits of the left paw were treated with 10 μL of 2‐octyl‐cyanoacrylate (Dermabond, Ethicon, LLC, San Lorenzo, Puerto Rico) immediately after amputation. Digits of the right paw were left untreated as controls. Dermabond was applied only to the distal tip of the digit, and digits were allowed to dry for 1 min. Digits were collected at specified time points for histological analysis. All experiments were performed in accordance with the standard operating procedures approved by the Institutional Animal Care and Use Committee of Tulane University Health Sciences Center. Administration of Hypoxyprobe™‐1 Plus (Hypoxyprobe, Burlington, MA) was per the manufacturer's instructions. Briefly, Hypoxyprobe was administered via intraperitoneal injection at a dosage of 60 mg/kg. Mice were sacrificed and digits harvested and fixed in Z‐fix after 45 min as previously described (Sammarco et al. 2014).

### Hyperbaric oxygen treatment

Following digit amputation, mice received HBOT on consecutive days during the course of regeneration. Mice with and without Dermabond treatment were placed in the hyperbaric chamber (Baromedical Research Institute, Van Meter & Associates, Harvey, LA) and received 90 min of 100% oxygen at 2.4 ATA twice a day with an interval of 8 h between each treatment. Compression and decompression of the chamber were executed at 2 psi/min. Control groups with and without Dermabond did not receive HBOT.

### Histology and immunofluorescence

Digits were fixed overnight in zinc buffered formalin (Anatech, Battle Creek, MI). Bone was decalcified for 8 h in a formic acid based decalcifier (Decal I, Surgipath, Richmond, IL). Samples were processed for paraffin embedding using a Leica TP 1020 Processor and 4 μm sections were obtained using a Leica RM2255 microtome (Leica, Buffalo Grove, IL). Sections were deparaffinized with xylene and rehydrated through a series of graded ethanols. Mayer's H&E Y (Sigma‐Aldrich, St Louis, MO) staining was executed as per the manufacturer's instructions. Mallory trichrome stained sections were carried out according to the manufacturer's instructions (American MasterTech, Lodi, CA). Coverslips were mounted with Permount mounting medium (Fisher Scientific, Waltham, MA). To identify Dermabond, slides were prepared using petroleum ether in place of xylene during processing, followed by staining with 0.5% Oil Red O in isopropanol according to the protocol previously outlined (Galil et al. 1984).

For immunohistochemistry, tissue sections were rehydrated through a graded series of ethanols, washed in phosphate‐buffered saline (PBS), blocked with a serum‐free protein block (Dako, Carpinteria, CA) and incubated with primary antibody overnight at 4°C. Primary antibodies used were rabbit anti‐mouse cathepsin K (Abcam, Cambridge, UK), rabbit anti‐mouse cytokeratin 5 (Abcam, Cambridge, UK), and rabbit anti‐mouse Ki67 (Abcam, Cambridge, MA). Goat anti‐rabbit secondary antibody conjugated to Alexa Fluor 488 or Alexa Fluor 568 (Invitrogen, Carlsbad, CA) was applied for 45 min at room temperature. Slides were washed with PBS and stained with DAPI (Invitrogen), and coverslips were mounted with Prolong Gold (Invitrogen). To detect Hypoxyprobe, the primary antibody anti‐Hypoxyprobe‐1 Plus conjugated to fluorescein isothiocyanate (FITC) (Hypoxyprobe) was used. Amplification of the probe was performed with a rabbit anti‐FITC secondary conjugated to horseradish peroxidase (Dako) and converted to fluorescent signal with a tyramide substrate according to the manufacturer's instructions (TSA kit #14, Invitrogen).

Brightfield images of histological sections were obtained using a 10× or 20× objective on an Olympus BX60 upright microscope equipped with an Olympus DP72 camera. Fluorescent micrographs were captured using a 10×, 20× or 60× objective on an Olympus BX61 fluorescence deconvolution microscope. Cell counts were performed using masking subsampling of positive fluorescent areas in Slidebook imaging software (Intelligent Imagine Innovations, Denver, CO). A total area of 488 or 568 nm fluorescent signals was calculated and normalized to total DAPI area. A representative section of each sample was imaged for cell count.

### Analysis of bone morphology and polarized light microscopy

The type of new bone growth was determined with picrosirius red staining and polarized light microscopy (Rich & Whittaker 2005; Shapiro 2008). Paraffin sections were deparaffinized with xylene and rehydrated through a graded series of ethanols. Slides were stained with picrosirius red (0.5 g sirius red F3B in 500 mL saturated aqueous picric acid, Sigma‐Aldrich) for 1 h and washed briefly in acidified water (5 mL glacial acetic acid in 1 L of water). Slides were dehydrated and mounted with Permount mounting medium (Fisher Scientific). Polarized light microscopy was carried out on an Olympus BX60 upright microscope equipped with filters to provide circularly polarized illumination according to published methods (Rich & Whittaker 2005).

### Micro‐computed tomography and X‐ray

Micro‐CT images were acquired using a VivaCT 40 (Scanco Medical AG, Brüttisellen, Switzerland) at 1000 projections per 180° with a voxel resolution of 10 μm^3^, and energy and intensity settings of 55 kV and 145 μA. Integration time for capturing the projections was set to 380 msec using continuous rotation. Images were segmented using the BoneJ (Doube et al. 2010) (Version 1.2.1) Optimize Threshold Plugin for ImageJ (Version 1.48c). Changes in bone volume were quantified using the BoneJ Volume Fraction Plugin for ImageJ and bone volume was normalized to total bone volume at DPA 0; 3D renderings of μCT scans were created using the 3D viewer plugin for ImageJ. X‐ray analysis was performed using the Kodak In Vivo Imaging System FX (Kodak Molecular Imaging Systems, New Haven, CT) and the Carestream Molecular Imaging software (Carestream Health Inc., Toronto, Canada). X‐rays were gathered at 35 kVp with a 30 sec exposure time, a 20 mm field of view and an F‐stop of 8. Final images were compiled using Adobe Photoshop CS4.

## Supporting information


**Figure S1**. (A) H&E staining of a control digit at DPA 12 shows the histology of the blastema in a control digit. This area was isolated and stained for collagen I (B) and III (C). (B) Whole‐mount staining with anti‐collagen I (Col I, red) and DAPI (blue) of the area delineated by the dotted outline in (A) shows that Col I is most evident in the outer perimeter of the blastema. (C) Whole‐mount staining with anti‐collagen III (Col III, red) and DAPI (blue) of the area delineated by the dotted outline in (A) shows increased Col III staining in the blastema compared to Col I. Projection image of 100 μm z‐stack with Zeiss confocal laser scanning system. Scale bars 100 μm. Red, Col I or Col III; blue, nuclei.

**Figure S2**. Oxygen profiling of the bone marrow and connective tissue in control and Dermabond‐treated using Hypoxyprobe (<1.3% oxygen) indicates relatively hypoxic microenvironments in the connective tissue at DPA 7 in Dermabond‐treated digits compared to controls. Hypoxyprobe+ area was normalized to total cellular area. Oxygen profiling of the bone marrow shows no statistical difference between control and Dermabond‐treated digits at DPA 3, 5, or 7. The anti‐Hypoxyprobe stained cells were selected and plotted as cell counts versus DAPI staining. Percentages of cells are shown in the bar graph. Error bars represent SEM. ^#^ *P* < 0.05. *N* = 5 digits per time point.
