# Supplementary figures and images for: Epidermal closure regulates histolysis during mammalian (Mus) digit regeneration
Source: Regeneration (Oxf). 2015 Jun 9;2(3):106–19. doi: 10.1002/reg2.34 (PMC4895321; doi:10.1002/reg2.34)

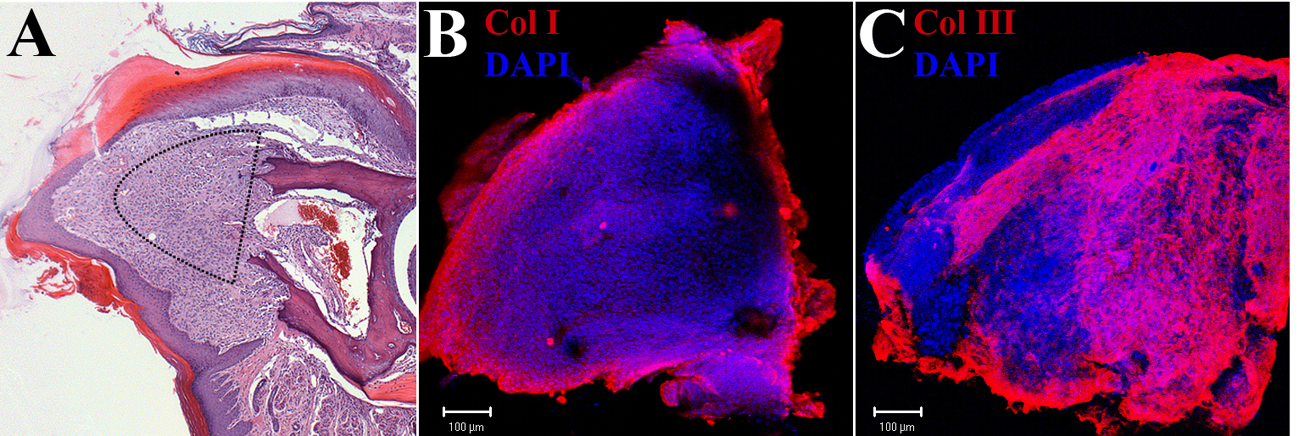

Supplement: Supplementary file 1 — Figure S1. (A) H&E staining of a control digit at DPA 12 shows the histology of the blastema in a control digit. This area was isolated and stained for collagen I (B) and III (C). (B) Whole‐mount staining with anti‐collagen I (Col I, red) and DAPI (blue) of the area delineated by the dotted outline in (A) shows that Col I is most evident in the outer perimeter of the blastema. (C) Whole‐mount staining with anti‐collagen III (Col III, red) and DAPI (blue) of the area delineated by the dotted outline in (A) shows increased Col III staining in the blastema compared to Col I. Projection image of 100 μm z‐stack with Zeiss confocal laser scanning system. Scale bars 100 μm. Red, Col I or Col III; blue, nuclei. [file REG2-2-106-s001.tif]

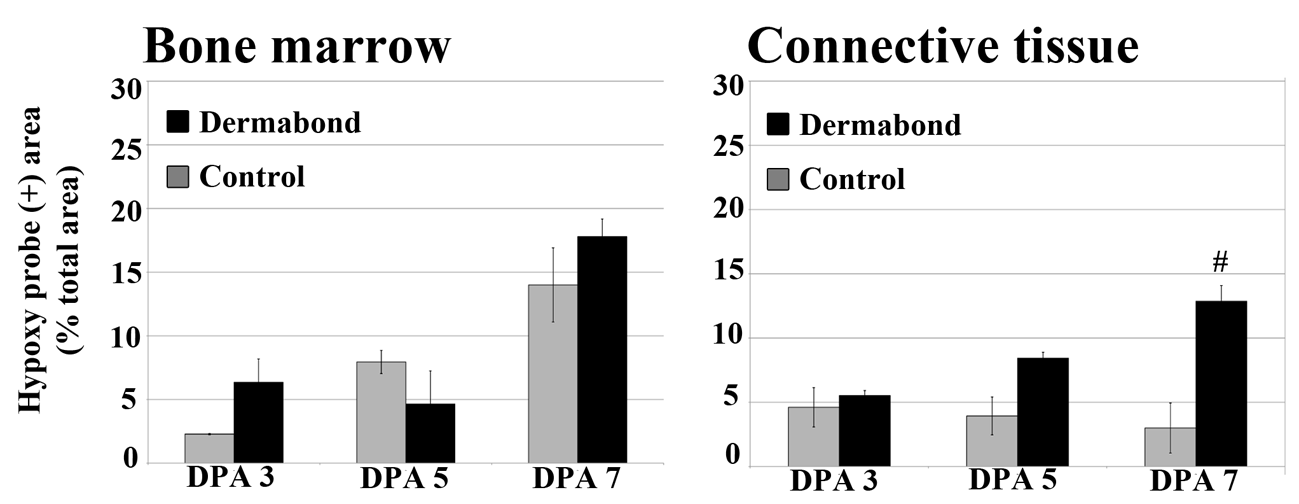

Supplement: Supplementary file 2 — Figure S2. Oxygen profiling of the bone marrow and connective tissue in control and Dermabond‐treated using Hypoxyprobe (<1.3% oxygen) indicates relatively hypoxic microenvironments in the connective tissue at DPA 7 in Dermabond‐treated digits compared to controls. Hypoxyprobe+ area was normalized to total cellular area. Oxygen profiling of the bone marrow shows no statistical difference between control and Dermabond‐treated digits at DPA 3, 5, or 7. The anti‐Hypoxyprobe stained cells were selected and plotted as cell counts versus DAPI staining. Percentages of cells are shown in the bar graph. Error bars represent SEM. # P < 0.05. N = 5 digits per time point. [file REG2-2-106-s002.tif]
